# Supplementary material for: Intrinsically disordered regions regulate both catalytic and non-catalytic activities of the MutLα mismatch repair complex
Source: Nucleic Acids Res. 2018 Dec 12;47(4):1823–35. doi: 10.1093/nar/gky1244 (PMC6393296; doi:10.1093/nar/gky1244)
Supplement: Supplementary Data [file gky1244_supplemental_files.docx]

**
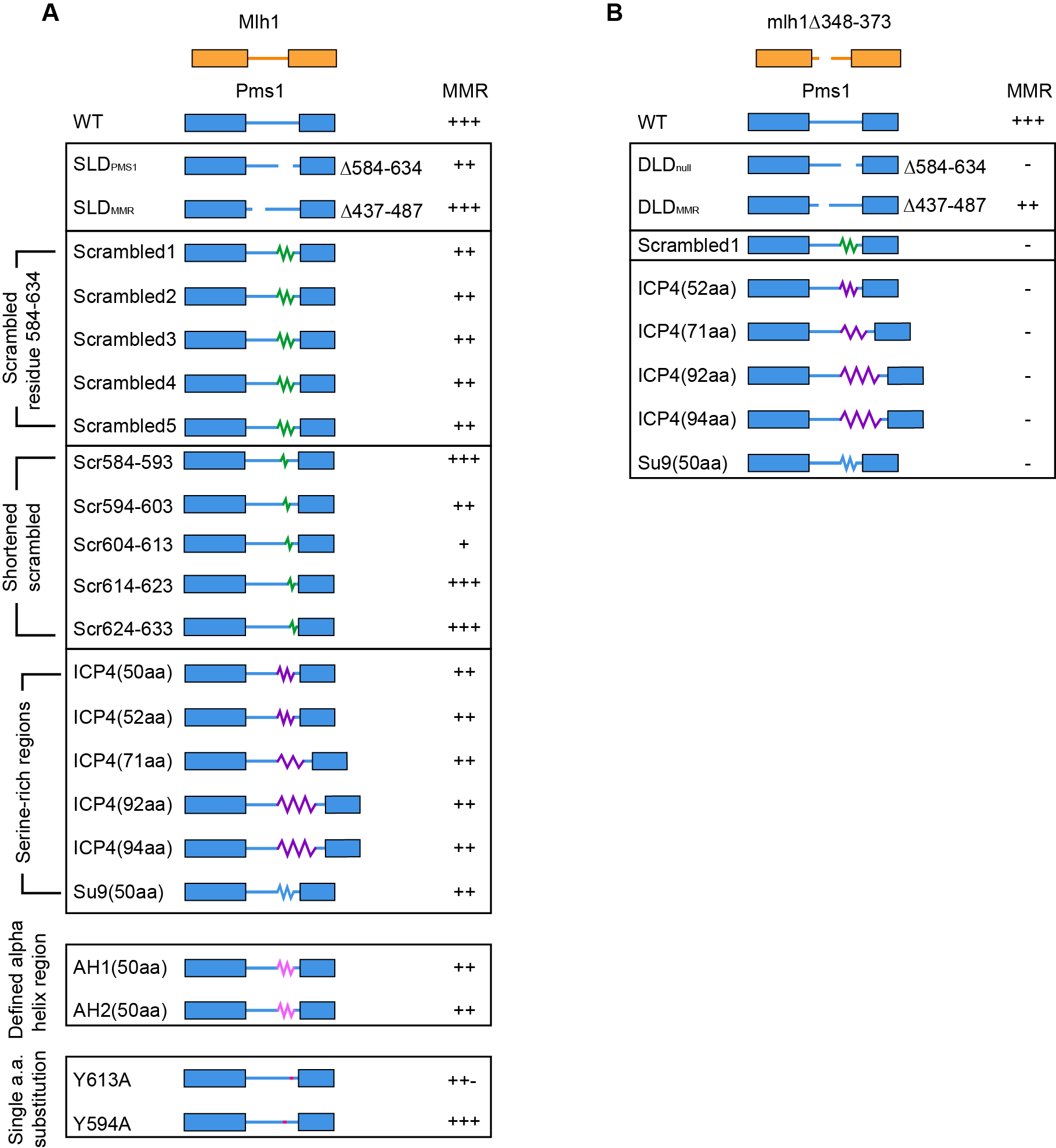
**

**Figure S1.** (A) Schematic of specific sequences that replace the IDRs in Pms1, followed by the mutator phenotype conferred by the indicated alleles in the *MLH1* strain background. +++ indicates a wild-type mutation rate, ++, ++- and + indicate hypomorph phenotypes, and – indicates a null phenotype. See text and ***Supplementary Table S1*** and ***Supplementary Table S4*** for quantitative data and detailed description of the specific sequences. (B) Analysis of linker alleles presented in panel A in the *mlh1-Δ348-373* background.


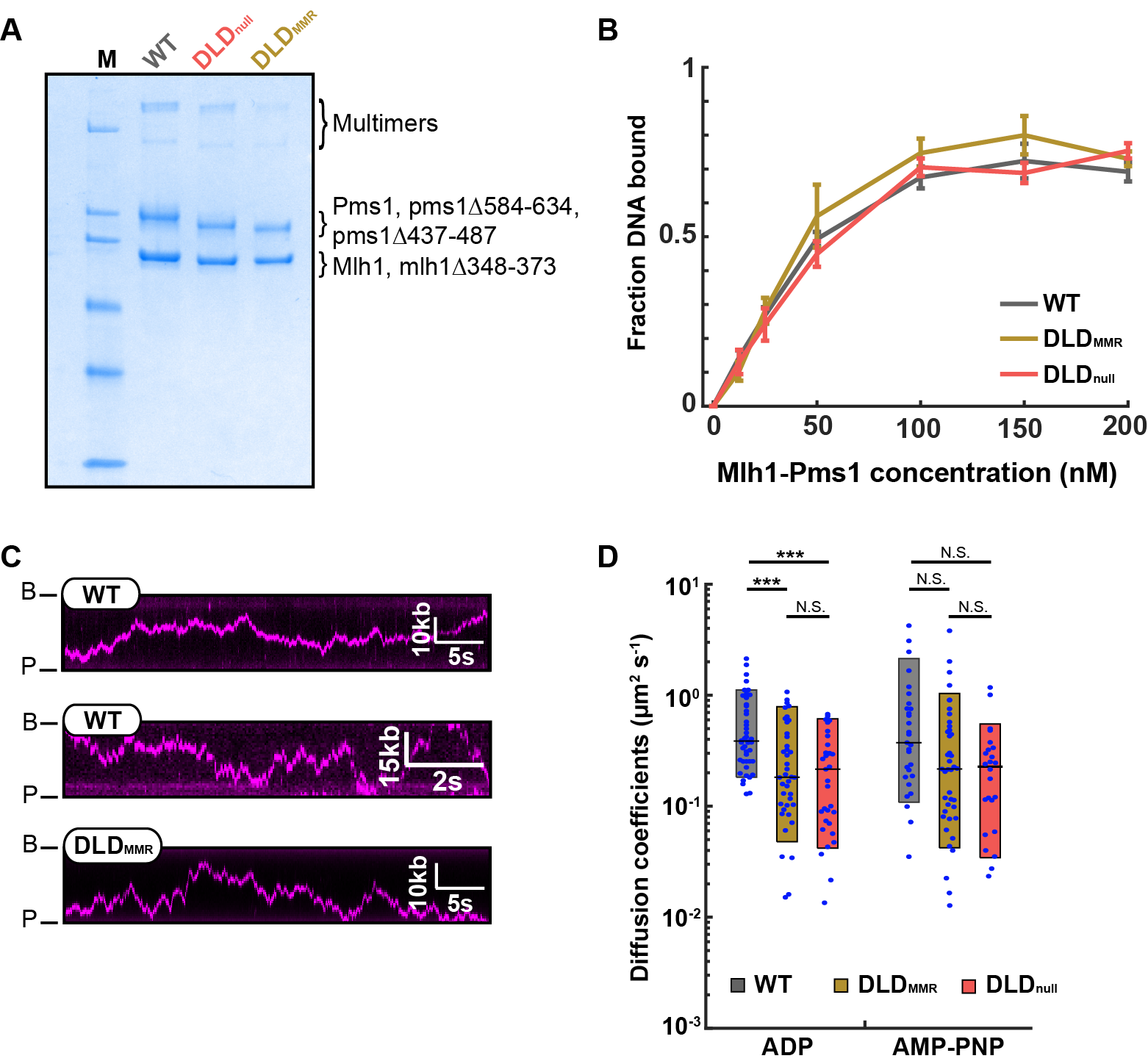


**Figure S2.** (A) SDS-PAGE analysis (8% Coomassie blue R250 stained gel) of purified wild-type (WT) Mlh1-FLAG-Pms1, DLD_null_ (mlh1Δ348-373-FLAG-pms1Δ584-634), and DLD_MMR_ (mlh1Δ348-373-FLAG-pms1Δ438-487). (B) Analysis of DNA binding in the absence of nucleotide. For each complex, DNA binding was analyzed by filter binding. Mlh1-Pms1 variants were included at final concentrations of 12.5 nM, 25 nM, 50 nM, 100 nM, 150 nM and 200 nM in buffer containing 25 mM NaCl. DNA binding was quantified by scintillation counting. Four replicates were averaged; error bars indicate the SD. (C) Representative kymographs of WT and DLD_MMR_ complexes loaded at 50 mM NaCl and imaged at 150 mM NaCl (top and bottom), and WT Mlh1-Pms1 loaded and imaged at 150 mM NaCl (middle). These images show that the proteins are freely diffusing on DNA. Complexes that were loaded at low or high NaCl concentration were indistinguishable in the single-molecule assays. B and P indicate barrier and pedestals, respectively. (D) Diffusion coefficients of the four Mlh1-Pms1 complexes with ADP or AMP-PNP. The black bar in the box plot represents the median of the distribution. * *P*-values <0.05, ** *P*-value < 0.01, and *** *P* value < 0.005. N.S. indicates p > 0.05.

**
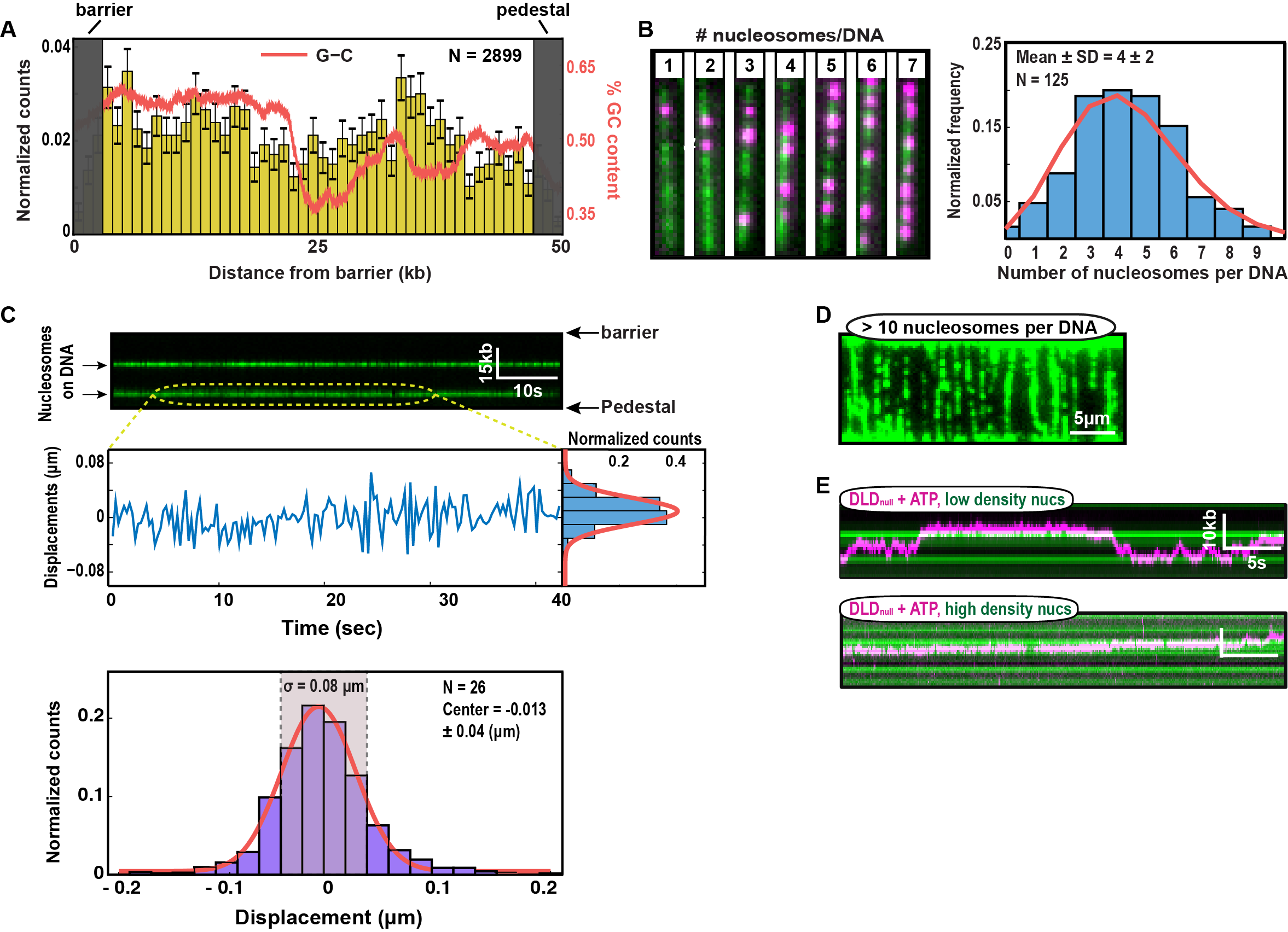
**

**Figure S3.** (A) Distribution of human nucleosomes on DNA curtains indicates a weak preference for GC-rich sequences. Error bars were generated by bootstrap analysis. The % GC content for a 2 kbp sliding window is shown in the red line. (B) Representative images of various numbers of nucleosomes per DNA (left), and a histogram of frequency of nucleosome deposition on DNA fitted to a Poisson distribution (red line) with the mean of the data (right). (C) Definition of ‘nucleosome zone' for bypass analysis. To determine the spatial resolution, a distribution of the net displacement of single nucleosomes was fit to a Gaussian distribution (red line). For analyzing single nucleosome bypass frequencies, the nucleosome zone was defined as a three-sigma region surrounding the mean nucleosome position (bottom). (D) A fluorescent image of double-tethered DNA curtain with > 10 nucleosomes per DNA. Nucleosomes were labeled with anti-HA antibody conjugated QDs (green). The position of each nucleosome cannot be determined due to overlapping fluorescent nucleosome signals. (E) Representative kymograph of DLD_null_ on different nucleosome density substrate in the presence of ATP.

**
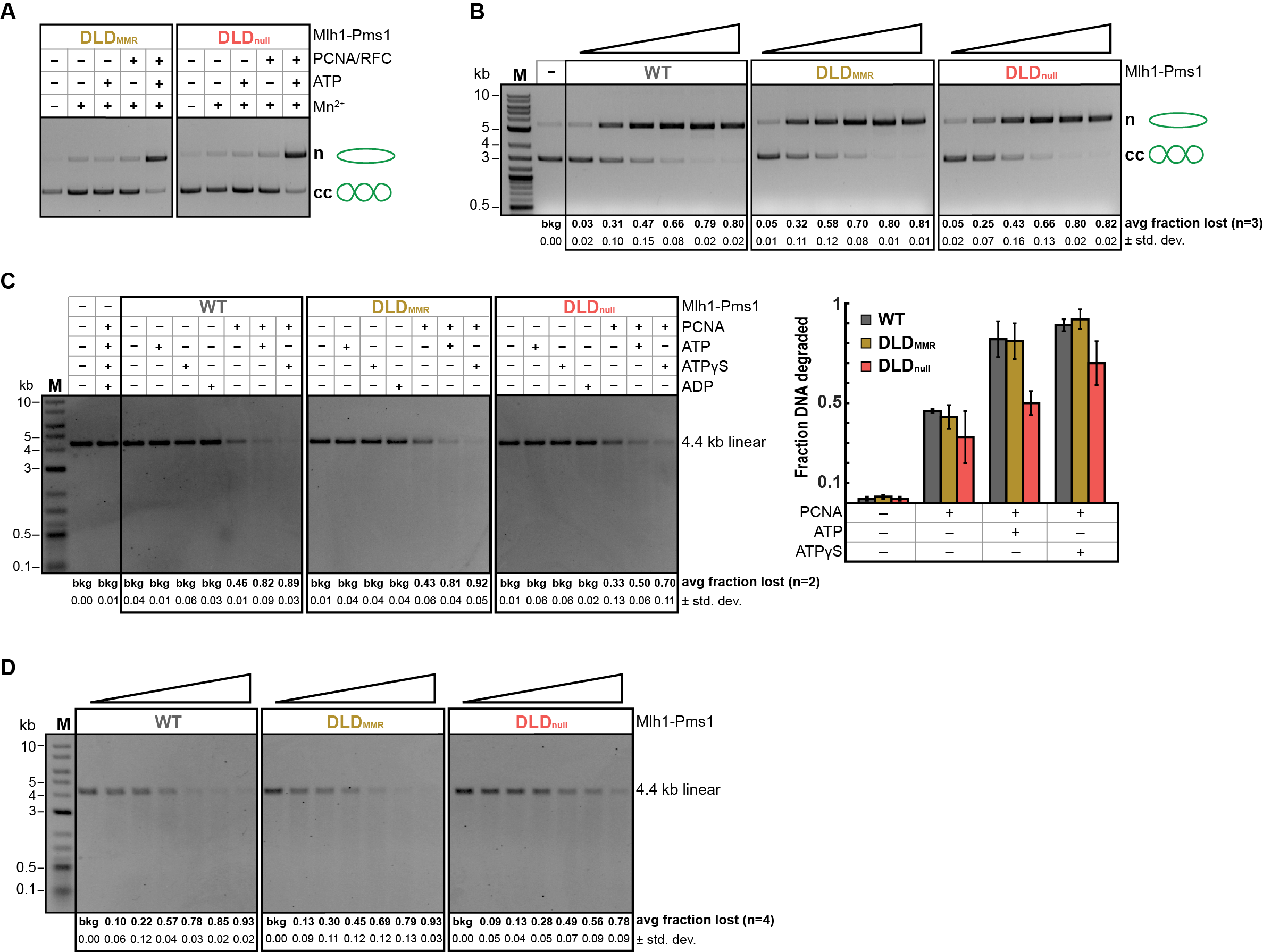
**

**Figure S4.** (A) Endonuclease activity on a closed circular DNA in the presence (+) or absence (-) of MnSO_4_, ATP, and yeast RFC/PCNA. Where + is indicated, the concentration of MnSO_4_ was 2.5 mM, ATP was 0.5 mM, RFC and PCNA were each 500 nM. The final concentration of Mlh1-Pms1 variants was 100 nM. All variants were analyzed side-by-side with WT as a control (***Figure 4A***). (B) Gel for data in ***Figure 4A*** right panel. Mlh1-Pms1 variants were titrated from 0-200 nM in the presence of 2.5 mM MnSO_4_, 0.5 mM, ATP, 500 nM RFC, and 500 nM PCNA. All variants were analyzed side-by-side with WT as a control. Final image is arranged for simplicity. Quantification from three separate experiments is shown below the gel. (C) Left panel, where indicated, the final concentration of nucleotide is 0.5 mM. The amount of substrate lost was quantified and expressed as a fraction by comparing to the average of lanes 2-3. The average fraction lost was calculated from duplicate experiments. The standard deviation between two independent experiments is expressed below the gel. All variants were analyzed side-by-side with WT as a control. Final image is arranged for simplicity. Right panel, analysis of endonuclease activity of wild-type and mutant Mlh1-Pms1 complexes (100 nM) on linear DNA. Reactions contain 500 nM PCNA and 0.5 mM ATP or ATPγS as indicated. 5 mM MnSO_4_ is included in all reactions. (D) Gel for data reported in ***Figure 4B***. The amount of substrate lost was quantified and expressed as a fraction by comparing to lane 2, 9, or 16 for each variant. The average fraction lost and SD were calculated from four replicates (SD expressed below the gel). All variants were analyzed in a single gel. Final image is arranged for simplicity.

**Supplementary Table S1.** ***pms1* linker arm insertions are unable to rescue *pms1Δ584-634* MMR defects**

| relevant genotype | mutation rate (x 10^-7^) (95% CI) | *n* | relative to wild type |
| --- | --- | --- | --- |
| *pms1∆* (EAY3097) | 25,700 (15,400-36,700) | 45 | 7,930 |
| *PMS1* | 3.24 (2.75-4.32) | 40 | 1.0 |
| *pms1∆*_584-634_ | 308 (230-452) | 24 | 95 |
| *pms1_50-scramble1_* | 332 (213-414) | 20 | 103 |
| *pms1_50-scramble2_* | 238 (184-338) | 20 | 74 |
| *pms1_50-scramble3_* | 335 (303-405) | 20 | 104 |
| *pms1_50-scramble4_* | 413 (339-637) | 20 | 127 |
| *pms1_50-scramble5_* | 520 (357-615) | 20 | 161 |
| *pms1_40-alpha-helix1_* | 378 (214-505) | 20 | 117 |
| *pms1_40-alpha-helix2_* | 465 (335-645) | 20 | 144 |
| *pms1_50-SRR-1_* | 436 (391-575) | 20 | 135 |
| *pms1_52-SRR_* | 315 (210-444) | 20 | 97 |
| *pms1_71-SRR_* | 413 (318-592) | 20 | 128 |
| *pms1_92-SRR_* | 421 (314-639) | 20 | 130 |
| *pms1_94-SRR_* | 514 (414-745) | 20 | 159 |
| *pms1_50-SRR-2_* | 229 (120-340) | 20 | 71 |
| *pms1_scramble584-593_* | 33.4(12.1-58.3) | 15 | 10 |
| *pms1_scramble594-603_* | 286(157-428) | 15 | 88 |
| *pms1_scramble604-613_* | 1,180(870-1,360) | 15 | 365 |
| *pms1_scramble614-623_* | 28.6(13.8-42.6) | 15 | 9 |
| *pms1_scramble624-633_* | 26.3(16.7-30.8) | 15 | 8 |
| *pms1_Y613A_* | 89(55-128) | 20 | 27 |
| *pms1_Y594A_* | 22.7(8.8-32.8) | 15 | 7 |
| *mlh1∆, pms1∆* (EAY1365) | 13,400 (9,330-15,400) | 30 | 4,570 |
| *MLH1, PMS1* | 2.94 (2.2-4.1) | 20 | 1.0 |
| *mlh1∆_348-373_, PMS1* | 206 (118-256) | 20 | 70 |
| *mlh1∆_348-373_, pms1∆*_584-634_ | 8,420 (4,460-10,300) | 20 | 2,860 |
| *mlh1∆_348-373_, pms1∆*_437-487_ | 429 (393-857) | 20 | 146 |
| *mlh1∆_348-373_, pms1_50-scramble1_* | 13,100 (9,280-17,400) | 20 | 4,450 |
| *mlh1∆_348-373_, pms1_52-SRR_* | 3,000 (2,150-7,470) | 20 | 1,020 |
| *mlh1∆_348-373_, pms1_71-SRR_* | 3,858 (3,260-7,150) | 20 | 1,320 |
| *mlh1∆_348-373_, pms1_92-SRR_* | 5,870 (5,100-7,330) | 20 | 2,000 |
| *mlh1∆_348-373_, pms1_50-SRR-2_* | 3,430 (2,890-6,180) | 20 | 1,170 |
| *Mlh1, pms1_Mlh1 linker_* | 13,700 (9,580-19,700) | 15 | 4,670 |
| *mlh1_Pms1 linker_, Pms1* | 7,680 (4,170-11,700) | 15 | 2,610 |
| *mlh1_Pms1 linker_, pms1_Mlh1 linker_* | 8,330 (3,130-20,400) | 15 | 2.830 |

The indicated *mlh1* and *pms1* alleles were tested in the *lys2::insE-A_14_* reversion assay, and Lys^+^ reversion rates (CI, confidence interval) were calculated as described in the *Materials and Methods*. n, number of independent measurements. The *PMS1* and *pms1* alleles were expressed from the native *PMS1* promoter in pRS413 derived *ARS-CEN HIS3* plasmids. The *MLH1* and *mlh1* alleles were expressed from the native *MLH1* promoter in pRS415 derived *ARS-CEN* *LEU2* plasmids. These plasmids (***Supplementary Table S4***) were transformed into EAY3097 (*pms1∆*) and EAY1365 (*mlh1∆, pms1∆*) strains.

**Supplementary Table S2. Nucleotide-dependent Mlh1-Pms1 diffusion coefficients**

| Nucleotide type | Protein type | 1D diffusion ± S.E.M.  (µm^2^ s^-1^) | Number of molecules | p-value  (relative to WT) | |
| --- | --- | --- | --- | --- | --- |
|  |  |  |  | t-test | K-S test |
| None | WT | 0.507 ± 0.07 | 56 | N/A | |
|  | DLD_MMR_ | 0.223 ± 0.04 | 41 | 0.1 | 0.01 |
|  | DLD_null_ | 0.078 ± 0.01 | 39 | 1.5x10^-6^ | 6.7x10^-9^ |
| ADP | WT | 0.584 ± 0.07 | 46 | N/A | |
|  | DLD_MMR_ | 0.303 ± 0.04 | 40 | 0.001 | 0.0007 |
|  | DLD_null_ | 0.376 ± 0.14 | 34 | 0.15 | 0.001 |
| AMP-PNP | WT | 0.753 ± 0.18 | 30 | N/A | |
|  | DLD_MMR_ | 0.451 ± 0.11 | 41 | 0.13 | 0.99 |
|  | DLD_null_ | 0.275 ± 0.05 | 26 | 0.02 | 0.07 |
| ATP | WT | 0.918 ± 0.10 | 59 | N/A | |
|  | DLD_MMR_ | 0.646 ± 0.10 | 50 | 0.069 | 0.007 |
|  | DLD_null_ | 0.171 ± 0.02 | 42 | 8.2x10^-8^ | 9.3x10^-13^ |

All data points were acquired in imaging buffer containing 150 mM NaCl.

**Supplementary Table S3. Probability of single nucleosome bypass by Mlh1-Pms1 complexes**

| Nucleotide type | Protein-Condition | Number of trajectories | Number of collisions | Probability ± std. dev. | P-value (relative to WT) | P-value (relative to DLD_MMR_) | Average number of collisions per trajectory ± std. dev. |
| --- | --- | --- | --- | --- | --- | --- | --- |
| Minus | WT | 31 | 1361 | 0.30 ± 0.003 | N/A |  | 44 ± 44 |
|  | DLD_MMR_ | 29 | 1166 | 0.18 ± 0.005 | 2.8x10^-11^ |  | 40 ± 29 |
|  | DLD_null_ | 27 | 1033 | 0.19 ± 0.002 | 6.1x10^-9^ | 0.54 | 38 ± 24 |
| ATP | WT | 34 | 1223 | 0.21 ± 0.007 | N/A |  | 35 ± 35 |
|  | DLD_MMR_ | 30 | 1572 | 0.063 ± 0.004 | 4.8x10^-28^ |  | 56 ± 60 |
|  | DLD_null_ | 30 | 1111 | 0.070 ± 0.002 | 1.9x10^-19^ | 0.08 | 37 ± 30 |

P-values are determined from fitting binary logistic regression relative to WT with same nucleotide condition.

**Supplementary Table S4. Plasmids used in this study**

| Plasmid | Relevant genotype | Vector type | Source |
| --- | --- | --- | --- |
| pRS413 |  | *ARS-CEN, HIS3* | (Christianson et al., 1992) |
| pRS415 |  | *ARS-CEN, LEU2* | (Christianson et al., 1992) |
| pEAA213 | *MLH1* | *ARS-CEN, LEU2* | (Plys et al., 2012) |
| pEAA526 | *mlh1∆_348–373_ (FLAG_499_)* | *ARS-CEN, LEU2* | (Plys et al., 2012) |
| pEAA238 | *PMS1* | *ARS-CEN, HIS3* | (Plys et al., 2012) |
| pEAA544 | *pms1Δ_437-487_ (HA_565_)* | *ARS-CEN, HIS3* | (Plys et al., 2012) |
| pEAA548 | *pms1Δ_584–634_ (HA_565_)* | *ARS-CEN, HIS3* | (Plys et al., 2012) |
| pEAA644 | *pms1_50-scramble1_* | *ARS-CEN, HIS3* | This study |
| pEAA645 | *pms1_50-scramble2_* | *ARS-CEN, HIS3* | This study |
| pEAA646 | *pms1_50-scramble3_* | *ARS-CEN, HIS3* | This study |
| pEAA647 | *pms1_50-scramble4_* | *ARS-CEN, HIS3* | This study |
| pEAA648 | *pms1_50-scramble5_* | *ARS-CEN, HIS3* | This study |
| pEAA649 | *pms1_40-alpha-helix1_* | *ARS-CEN, HIS3* | This study |
| pEAA650 | *pms1_40-alpha-helix2_* | *ARS-CEN, HIS3* | This study |
| pEAA651 | *pms1_50-SRR-1_* | *ARS-CEN, HIS3* | This study |
| pEAA652 | *pms1_52-SRR_* | *ARS-CEN, HIS3* | This study |
| pEAA653 | *pms1_71-SRR_* | *ARS-CEN, HIS3* | This study |
| pEAA654 | *pms1_92-SRR_* | *ARS-CEN, HIS3* | This study |
| pEAA655 | *pms1_94-SRR_* | *ARS-CEN, HIS3* | This study |
| pEAA656 | *pms1_50-SRR-2_* | *ARS-CEN, HIS3* | This study |
| pEAA657 | *pms1_full mlh1 Linker_* | *ARS-CEN, HIS3* | This study |
| pEAA658 | *mlh1_full pms1 Linker_* | *ARS-CEN, LEU2* | This study |
| pEAA659 | *pms1_Y613A_* | *ARS-CEN, HIS3* | This study |
| pEAA660 | *pms1_Y594A_* | *ARS-CEN, HIS3* | This study |
| pEAA661 | *pms1_scramble584-593_* | *ARS-CEN, HIS3* | This study |
| pEAA662 | *pms1_scramble594-603_* | *ARS-CEN, HIS3* | This study |
| pEAA663 | *pms1_scramble604-613_* | *ARS-CEN, HIS3* | This study |
| pEAA664 | *pms1_scramble614-623_* | *ARS-CEN, HIS3* | This study |
| pEAA665 | *pms1_scramble624-633_* | *ARS-CEN, HIS3* | This study |
| pEAE269 | *GAL1-MLH1(FLAG_499_)-VMA1-CBD* | *2μ,* *TRP1* | (Plys et al., 2012) |
| pEAE308 | *GAL1-mlh1∆_348–373_(FLAG_499_)-VMA1-CBD* | *2μ*, *TRP1* | (Plys et al., 2012) |
| pMH8 | *GAL10-PMS1* | *2μ,* *LEU2* | (Hall and Kunkel, 2001) |
| pEAE388 | *GAL10-pms1Δ_437-487_* | *2μ*, *LEU2* | This study |
| pEAE419 | *GAL10-pms1Δ_584–634_* | *2μ*, *LEU2* | This study |
| Plasmid constructs expressing pms1 proteins in which the specified amino acid sequences replace those deleted in *pms1Δ_584–634_.* | | | |
| Plasmid | insert | *amino acid sequence replacement* | |
| pEAA644 | *pms1_50-scramble1_* | *SSKSNKFGINSNKSLIDGKRNERFLMLDKLKNSEQIISTRESDSKYYETHI* | |
| pEAA645 | *pms1_50-scramble2_* | *ISKMSKILKKNIESSSGEYNKSFLNSEQRNSYSGTLINLKDDTEDIKFRHR* | |
| pEAA646 | *pms1_50-scramble3_* | *DTIERNGESIKLDFSESFKKSISTKSKLKNMESSIRYKIRGLLNDNYQSNH* | |
| pEAA647 | *pms1_50-scramble4_* | *NKSSQSKIGLRSRLYIGTMTENSSKSFEDIKKHSDSNILLYKNFRDEKINE* | |
| pEAA648 | *pms1_50-scramble5_* | *GGRNIKEINFKLIQIKKEDSLLSKSIDMRHTKSNFTNYESYSDKESRSSNL* | |
| pEAA649 | *pms1_40-alpha-helix1_* | *NSRKSEAAAKEAAAKEAAAKEAAAKEAAAKEAAAKEAAAKEAAAKQMSSII* | |
| pEAA650 | *pms1_40-alpha-helix2_* | *SMISQEAAAKEAAAKEAAAKEAAAKEAAAKEAAAKEAAAKEAAAKNKISSR* | |
| pEAA651 | *pms1_50-SRR-1_* | *SSSTSSDSGSSSSSSASSSSGSSSTSSDSGSSSSSSASSSSGSGTMKHGT* | |
| pEAA652 | *pms1_52-SRR_* | *RRSSSTSSDSGSSSSSSASSSSGSSSTSSDSGSSSSSSASSSSGSGTMKHGT* | |
| pEAA653 | *pms1_71-SRR_* | *SSSTSSDSGSSSSSSASSSSGSSSTSSDSGSSSSSSASSSSGSSSTSSDSGSSSSSSASSSSGSGTMKHGT* | |
| pEAA654 | *pms1_92-SRR_* | *SSSTSSDSGSSSSSSASSSSGSSSTSSDSGSSSSSSASSSSGSSSTSSDSGSSSSSSASSSSGSSSTSSDSGSSSSSSASSSSGSGTMKHGT* | |
| pEAA655 | *pms1_94-SRR_* | *RRSSSTSSDSGSSSSSSASSSSGSSSTSSDSGSSSSSSASSSSGSSSTSSDSGSSSSSSASSSSGSSSTSSDSGSSSSSSASSSSGSGTMKHGT* | |
| pEAA656 | *pms1_50-SRR-2_* | *MASTRVLASRLASQMAASAKVARPAVRVAXVSKRTIQTGSPLQTRAYSS* | |
| Plasmid constructs expressing pms1 proteins in which the specified amino acid sequences replace those deleted in the designated region | | | |
| pEAA661 | *pms1_scramble584-593_* | *ISKMSKILKK* | |
| pEAA662 | *pms1_scramble594-603_* | *NIESSSGEYN* | |
| pEAA663 | *pms1_scramble604-613_* | *KSFLNSEQRN* | |
| pEAA664 | *pms1_scramble614-623_* | *SYSGTLINLK* | |
| pEAA665 | *pms1_scramble624-633_* | *DDTEDIKFRH* | |

Full plasmid descriptions can be found in the Materials and Methods.
